# Supplementary material for: Easy Diameter Tuning of Silicon Nanowires with Low-Cost SnO2-Catalyzed Growth for Lithium-Ion Batteries
Source: Nanomaterials (Basel). 2022 Jul 28;12(15):2601. doi: 10.3390/nano12152601 (PMC9370699; doi:10.3390/nano12152601)
Supplement: Supplementary file 1 [file nanomaterials-12-02601-s001.zip › nanomaterials-1813010-supplementary.pdf]

Supplementary information

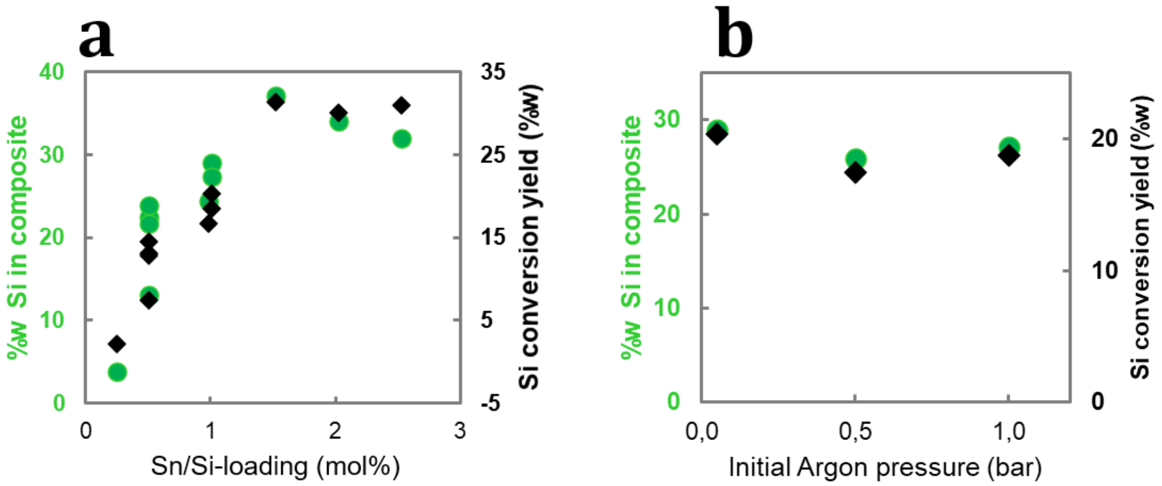

Figure S1. Influences of (**a**) the Sn/Si-loading and (**b**) the initial Argon pressure on the wt%Si in the composite and the Si conversion yield.

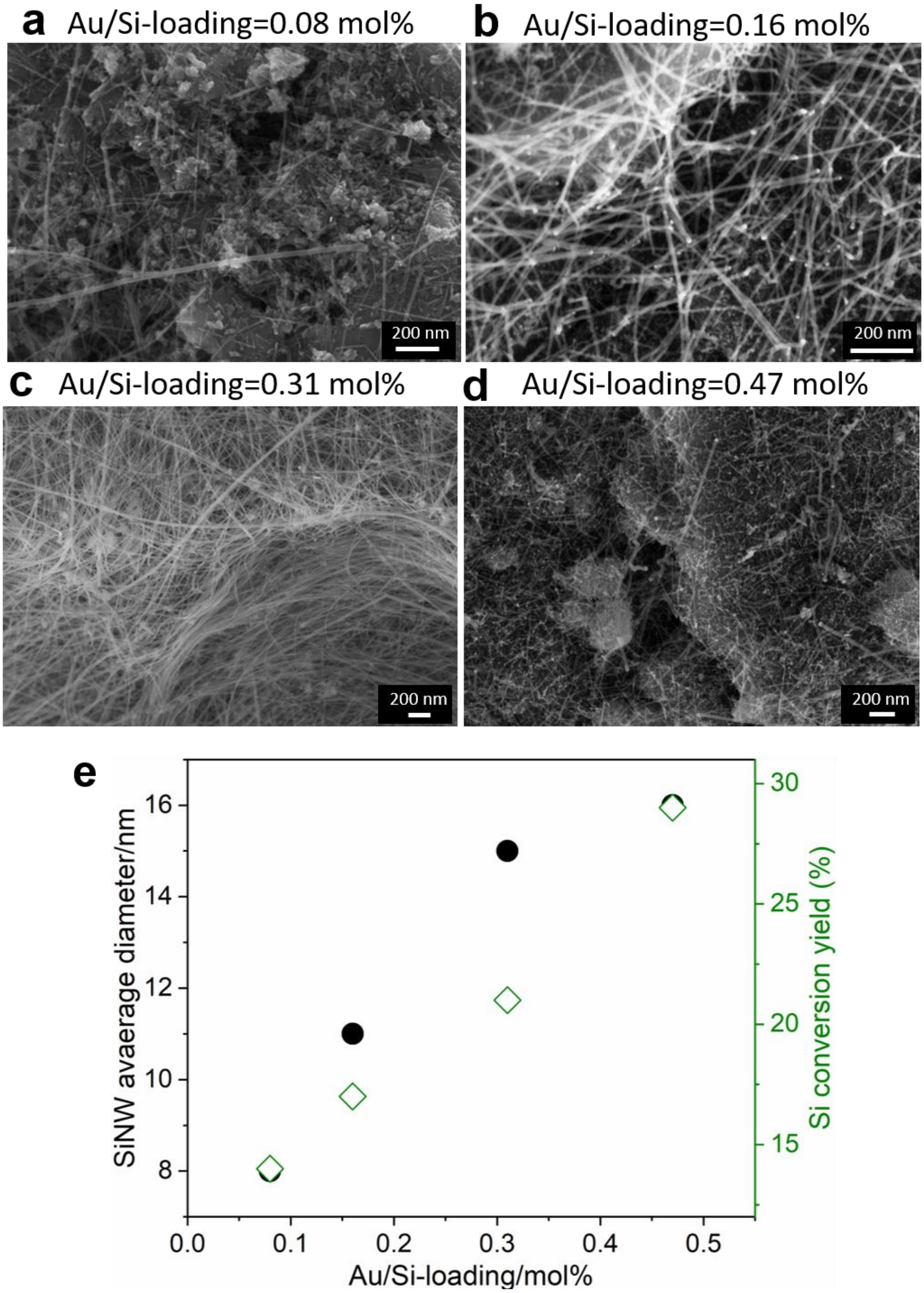

Figure S2. (**a**–**d**) SEM images of SiGt composites grown from gold catalysts (1.5-2nm gold nanoparticles) using Au/Si–loading 0.08 to 0.47 mol%. (**e**) Effect of Au/Si-loading on SiNW growth in SiGt: SiNW average diameters (dots) and Si conversion yield (diamonds) vs Au/Si-loading.

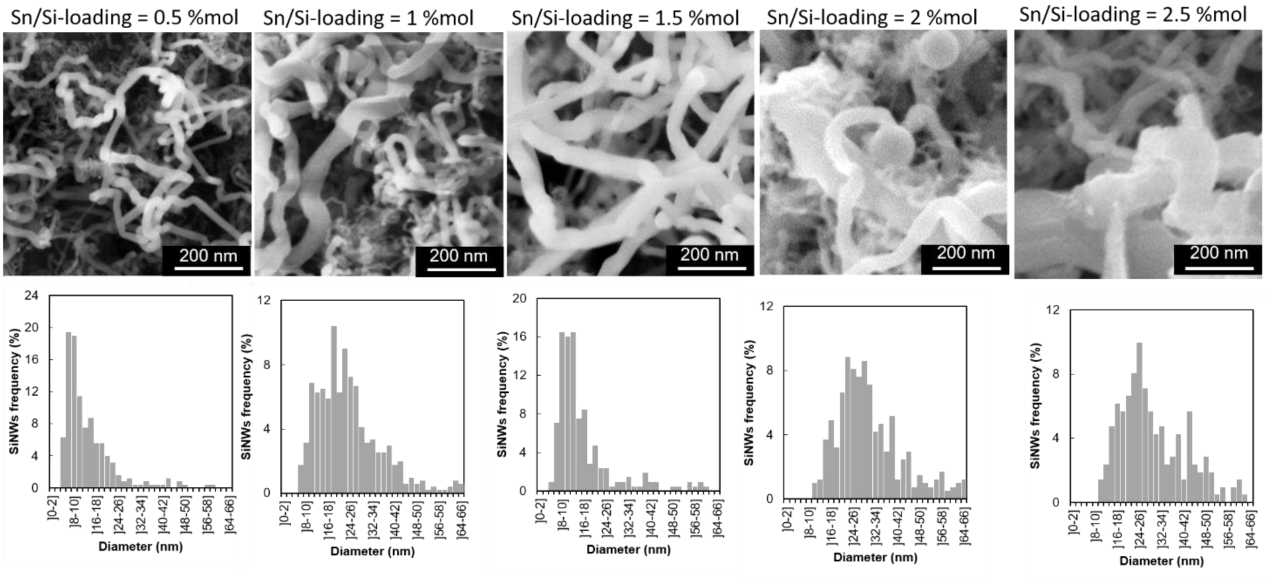

Figure S3. SEM images and corresponding diameter histograms for samples presented in Figure 2.

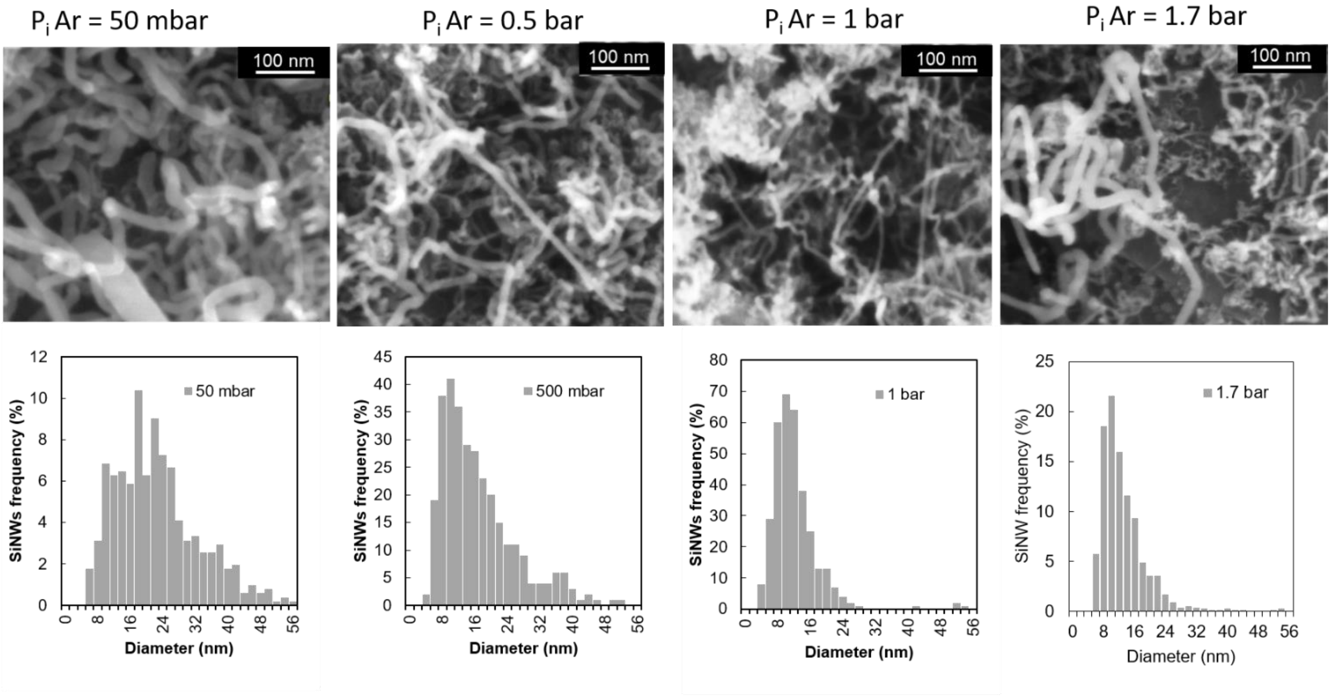

Figure S4. SEM images and corresponding diameter histograms for samples presented in Figure 3.

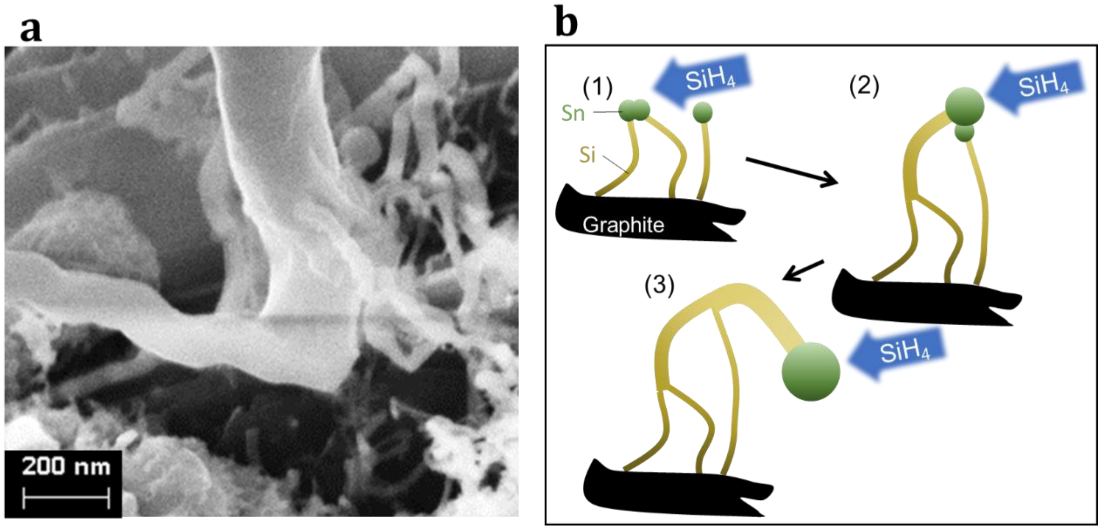

Figure S5. (**a**) SEM image show a silicon nanowire increasing its diameter after merging with smaller nanowires, (**b**) Proposed mechanism.

Table S1. Growth conditions.

Sample	Initial mass of graphite (mg)	Initial mass of SnO2 (mg)	Initial volume of phenylsilane/ di-phenylsilane (mL/mL)	Initial Sn/Si ratio (mol%)	Initial argon pressure in re-actor (bar)	Temperature and duration of growth (°C/ h)	Final quantity of composite (mg)
(1)	808.3	50	12/0	**0.5**	**0.05**	380 / 5	1097
(2)	802.5	50	8.4/2.4	**0.5**	**0.05**	380 / 5	947
(3) 30nm-SiGt	804.9	100	8.4/2.4	**1**	**0.05**	380 / 4	1074
(4) 30nm-SiGt	650	80	7/1.9	**1**	**0.05**	380 / 4.5	961
(5) 37nm-SiGt	808	200	8.4/2.4	**2**	**0.05**	380 / 4	1485
(6)	807	250	8.4/2.4	**2.5**	**0.05**	380 / 3	1585
(7)	652	80	7/1.9	**1**	**0.5**	380 / 5	948
(8) 14nm-SiGt	653	80	7/1.9	**1**	**1.0**	380 / 5	976
(9)	650	80	7/1.9	**1**	**1.7**	390 / 3	918

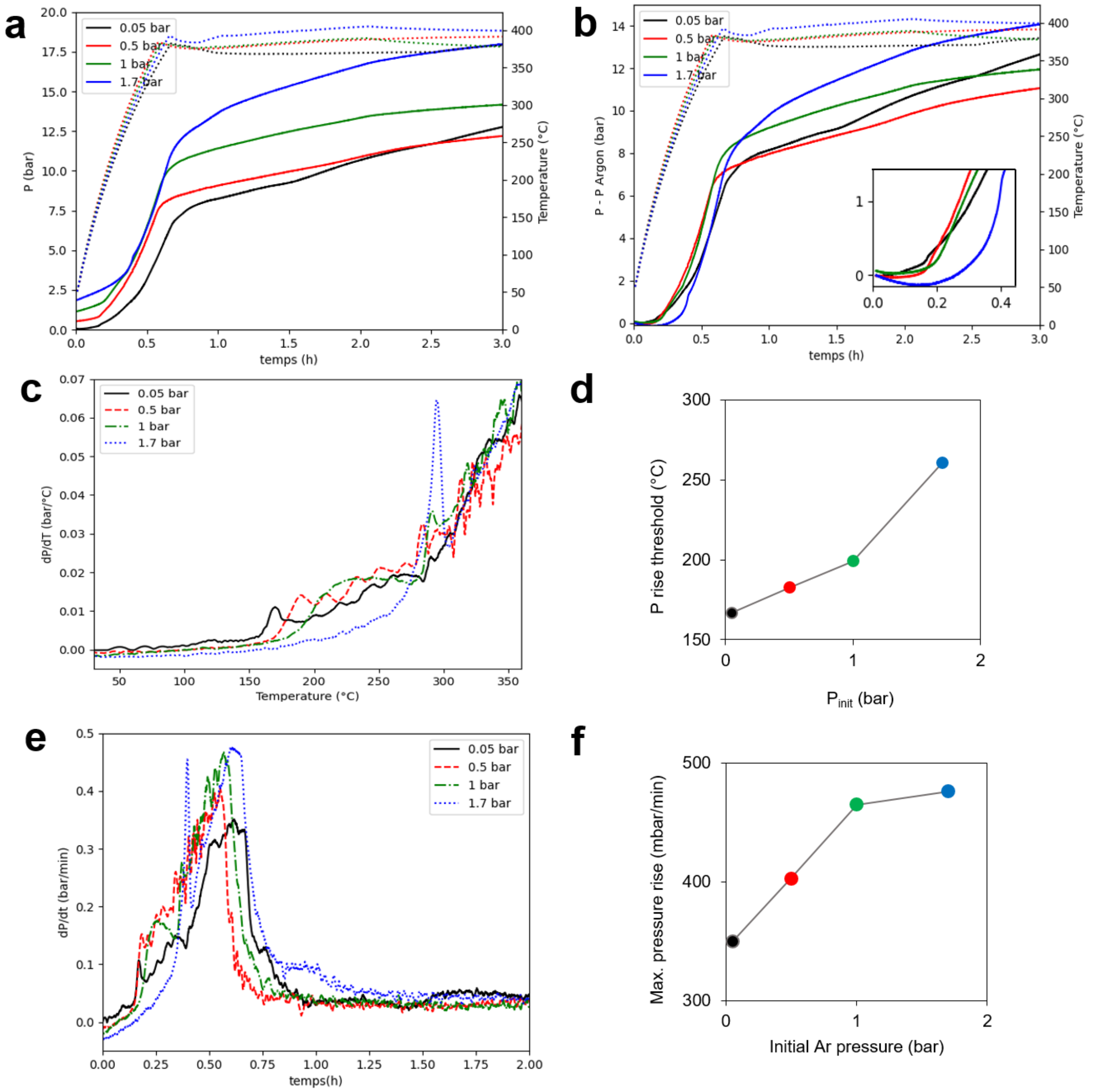

Figure S6. Pressure and temperature evolution during growth of SiGt at P_{init} 0.05 (black), 0.5 (red), 1 (green) and 1.7 bar (blue) for Sn/Si-loading = 1 wt%. (**a**) Pressure (lines) and temperature (dots) records vs time. (**b**) Argon-corrected pressure (line) and temperature (dots) vs time. The partial pressure of argon was computed according to the ideal gas law. Inset: enhanced view of the pressure rise. (**c**) Argon-corrected pressure derivative with temperature as a function of temperature for the growth process with an initial argon pressure of 0.05 (black), 0.5 (red), 1 (green) and 1.7 bar (blue). (**d**) Temperature at which the derivative attains 10mbar/°C vs P_{init}. (**e**) Argon-corrected pressure derivative with time vs time. (**f**) Maximal value of the argon-corrected pressure derivative with time as a function of P_{init}.

Table S2. Composition as measured by EDS and specific capacity Q_{sp} for SiGt samples tested in electrochemistry. The theoretical Q_{sp} is computed from the synthesis data, the experimental Q_{sp} is calculated from the charge of the 1st cycle.

	w% Gt	*w% Sn*	*w% Si*	*Theoretical Q_{sp} (mAh/g)*	*Experimental Q_{sp} (mAh/g)*
14nm-SiGt	67	2	31	1 375	1 086
30nm-SiGt	66	4	30	1 363	1 262
37nm-SiGt	55	11	29	1 527	1 427

Table S3. Comparison of the method for tuning SiNW diameter in the literature.

**Reference**	**SiNW diameter range**	**Synthesis method**	**Catalyst**	**Method for changing the SiNW diameter**
(1)	40-160 nm	PECVD	Au	Variation of Au film thickness
(2)	12-140 nm	CVD	Au	Variation of Au film thickness and SiH_4:HCl ratio
(3)	40-345 nm	CVD	Au	Variation of Au film thickness
(4)	50-100 nm	CVD	Au	Variation of Au nanoparticles sizes
(5)	10-50 nm	CVD	Au	Variation of SiH_4 partial pressure
(6)	5-30 nm	SFLS-CVD	Au	Variation of Au nanoparticles sizes
(7)	40-140 nm	PECVD	**SnO_2**	Time of H_2 plasma treatment and variation of H_2:SiH_4 ratio
(8)	60-220 nm	PPECVD	**Sn**	Variation of Sn film thickness
(9)	80-200nm	SFLS-CVD	**Sn**	Variation of initial Sn/Si ratio
This work	10-40 nm	CVD in closed reactor	**SnO_2**	Variation of Sn/Si ratio or addition of inert gas

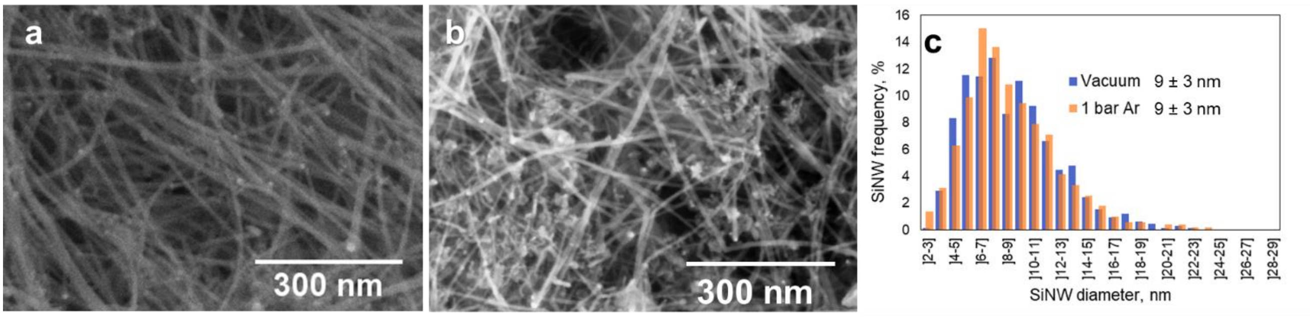

Figure S7. SEM images of SiNWs synthetized with 1-2 nm gold nanoparticles as catalyst, with the initial pressure of Argon being 0.05 bar (a) and 1 bar (b). (c) Frequencies of diameters measured for the two samples, with average and standard deviation values. Statistics calculated on at least 500 NWs on SEM images.

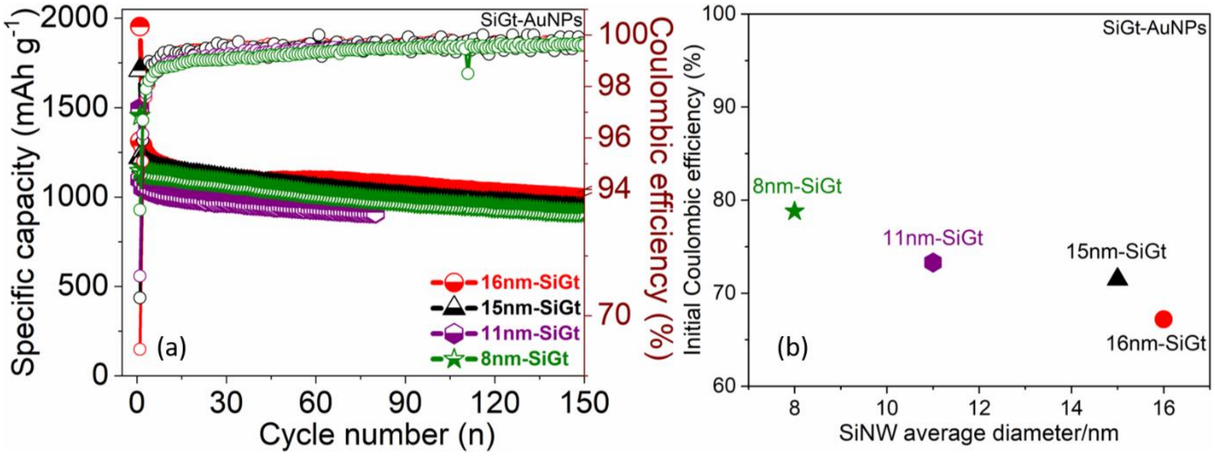

Figure S8. Electrochemical performance of SiGt-AuNPs anode tested in half-cell figuration vs Li metal. (a) Reversible charge/discharge capacity vs cycle number with corresponding Coulombic efficiency of SiGt-AuNPs anodes (having different average diameter of SiNW with respect to the Au-Si mol% loading) cycled at C/5 rate at room temperature in the 0.01-1.0 V vs Li^+/Li potential window and with 1 M $LiPF_6$ electrolyte in 1:1 (v/v ethylene carbonate (EC) and diethylene carbonate (DEC) with 2 wt % of vinylenecarbonate (VC) and 10 wt % of fluoroethylene carbonate (FEC). (b) Comparison of Colombic efficiency of SiGt-AuNPs anode versus average diameter of SiNW in the respective composite anodes.

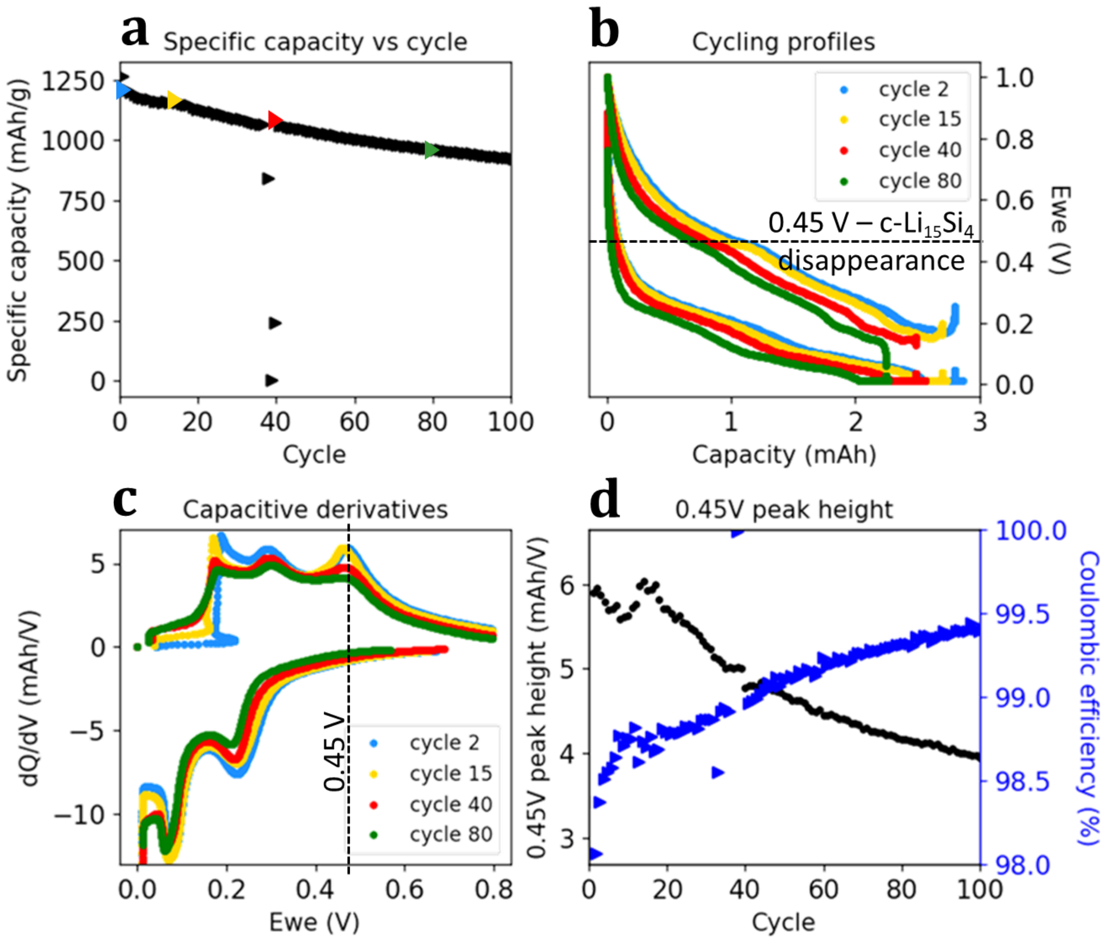

Figure S9. Correlation between the evolution of Coulombic efficiency and the c-$Li_{15}Si_4$ amount in 30nm-SiGt. (a) Specific capacity versus cycle number, (b) Cycling profiles for cycles 2, 15, 40, 80 showing a characteristic plateau at 0.45V for cycles 2 and 15, (c) Capacitive derivative of the corresponding profiles and (d) Comparison of the 0.45V peak with Coulombic efficiency evolution.

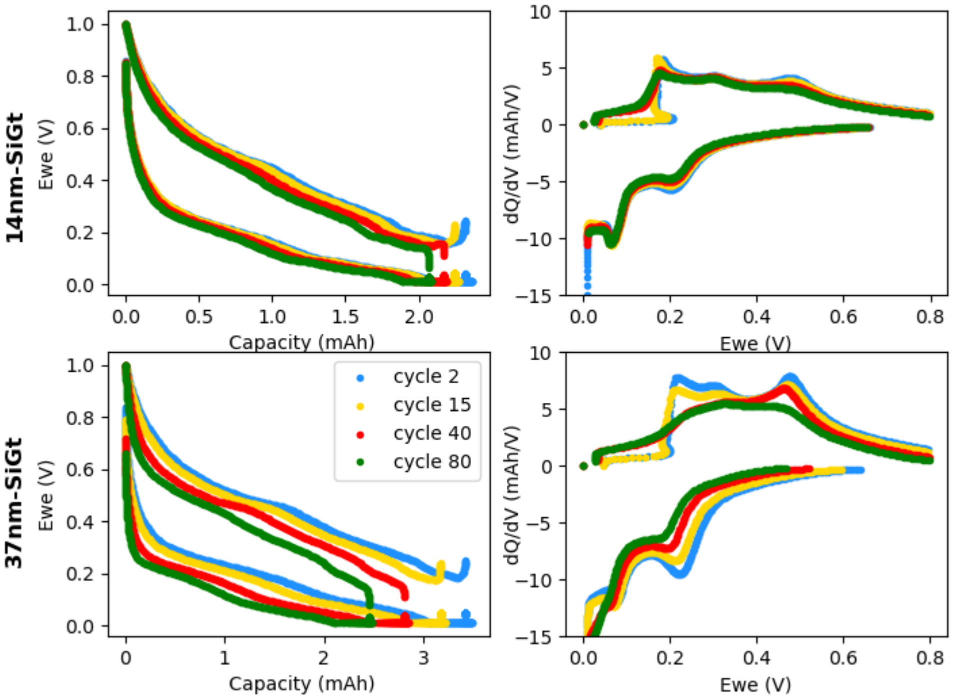

Figure S10. Cycling profiles (left) and capacitive derivatives (right) of 14nm-SiGt (upper panel) and 37nm-SiGt (downside panel).

References

1. Hamidinezhad, H. Thickness Effect of Catalyst Layer on Silicon Nanowires Morphology and Features. *Appl. Surf. Sci.* **2016**, *364*, 484–489. https://doi.org/10.1016/j.apsusc.2015.12.191.
2. Sharma, S.; Kamins, T. I.; Williams, R. S. Synthesis of Thin Silicon Nanowires Using Gold-Catalyzed Chemical Vapor Deposition. *Appl. Phys. A* **2005**, 80, 1225–1229. https://doi.org/10.1007/s00339-004-3155-3.
3. Gohier, A.; Laïk, B.; Pereira-Ramos, J.-P.; Cojocaru, C. S.; Tran-Van, P. Influence of the Diameter Distribution on the Rate Capability of Silicon Nanowires for Lithium-Ion Batteries. *J. Power Sources* **2012**, *203*, 135–139. https://doi.org/10.1016/j.jpowsour.2011.12.023
4. Langklotz, U.; Lein, T.; Schulze, C.; Weiser, M.; Krause, A.; Michaelis, A. Scalable Fabrication of Gold Nanoparticles with Adjustable Size Distribution as Catalytic Nuclei for the CVD Growth of Silicon Nanowires. *Appl. Surf. Sci.* **2020**, 502, 144203. https://doi.org/10.1016/j.apsusc.2019.144203
5. Dhalluin, F.; Desré, P. J.; den Hertog, M. I.; Rouvière, J.-L.; Ferret, P.; Gentile, P.; Baron, T. Critical Condition for Growth of Silicon Nanowires. Journal of Applied Physics 2007, 102 (9), 094906. https://doi.org/10.1063/1.2811935.
6. Kilian, S.; Adegoke, T.E.; Ahad, S.A.; Geaney, H., Kennedy, T.; Ryan, K.M., Temperature induced diameter variation of silicon nanowires via a liquid–solid phase transition in the Zn seed. *Chem. Commun.*, **2021**, 57, 12504-12507., https://pubs.rsc.org/en/content/articlelanding/2021/CC/D1CC04427C
7. Yu, L.; Alet, P.-J.; Picardi, G.; Maurin, I.; Cabarrocas, P. R. i. Synthesis, Morphology and Compositional Evolution of Silicon Nanowires Directly Grown on SnO2 substrates. *Nanotechnology* **2008**, *19*, 485605. https://doi.org/10.1088/0957-4484/19/48/485605.
8. Al-Taay, H. F.; Mahdi, M. A.; Parlevliet, D.; Jennings, P. Controlling the Diameter of Silicon Nanowires Grown Using a Tin Catalyst. *Mater. Sci. Semicond. Processing* **2013**, *16*, 15–22. https://doi.org/10.1016/j.mssp.2012.07.006.
9. Bogart, T.D.; Oka, D.; Lu, X.; Gu, M.; Wang, C.; Korgel, B.A. Lithium Ion Battery Peformance of Silicon Nanowires with Carbon Skin. *Acs Nano* **2013**, *8*, 915–922, https://doi.org/10.1021/nn405710w
